# Infant and Maternal Characteristics in Neonatal Abstinence Syndrome — Selected Hospitals in Florida, 2010–2011

**Published:** 2015-03-06

**Authors:** Jennifer N. Lind, Emily E. Petersen, Philip A. Lederer, Ghasi S. Phillips-Bell, Cria G. Perrine, Ruowei Li, Mark Hudak, Jane A. Correia, Andreea A. Creanga, William M. Sappenfield, John Curran, Carina Blackmore, Sharon M. Watkins, Suzanne Anjohrin

**Affiliations:** 1 Epidemic Intelligence Service, CDC; 2 Division of Nutrition, Physical Activity, and Obesity, National Center for Chronic Disease Prevention and Health Promotion, CDC; 3 United States Public Health Service Commissioned Corps; 4 Division of Reproductive Health, National Center for Chronic Disease Prevention and Health Promotion, CDC; 5 Division of Global HIV/AIDS, Center for Global Health, CDC; 6 Florida Department of Health; 7 University of Florida College of Medicine–Jacksonville; 8 Department of Community and Family Health, College of Public Health, University of South Florida; 9 Morsani College of Medicine, University of South Florida

Neonatal abstinence syndrome (NAS) is a constellation of physiologic and neurobehavioral signs exhibited by newborns exposed to addictive prescription or illicit drugs taken by a mother during pregnancy (1). The number of hospital discharges of newborns diagnosed with NAS has increased more than 10-fold (from 0.4 to 4.4 discharges per 1,000 live births) in Florida since 1995, far exceeding the three-fold increase observed nationally (1,2). In February 2014, the Florida Department of Health requested the assistance of CDC to 1) assess the accuracy and validity of using Florida’s hospital inpatient discharge data, linked to birth and infant death certificates, as a means of NAS surveillance and 2) describe the characteristics of infants with NAS and their mothers. This report focuses only on objective two, describing maternal and infant characteristics in the 242 confirmed NAS cases identified in three Florida hospitals during a 2-year period (2010–2011). Infants with NAS experienced serious medical complications, with 97.1% being admitted to an intensive care unit, and had prolonged hospital stays, with a mean duration of 26.1 days. The findings of this investigation underscore the important public health problem of NAS and add to current knowledge on the characteristics of these mothers and infants. Effective June 2014, NAS is now a mandatory reportable condition in Florida. Interventions are also needed to 1) increase the number and use of community resources available to drug-abusing and drug-dependent women of reproductive age, 2) improve drug addiction counseling and rehabilitation referral and documentation policies, and 3) link women to these resources before or earlier in pregnancy.

For this study, six hospitals in two Florida counties with high numbers of NAS births were identified using Florida’s hospital inpatient discharge data; of these, three hospitals were able to provide data needed for this investigation. Three data sources were used to identify infants with possible NAS: linked administrative data (Florida’s linked hospital inpatient discharge, birth certificate, and infant death certificate data), data collected through neonatal intensive care unit (NICU) admission logs, and inpatient pharmacy data. The linked administrative data selection criteria were maternal residency in Florida, nonadoption status of the infant, and birth of the infant at one of the three participating hospitals during 2010–2011. Infants with an *International Classification of Diseases, Ninth Revision, Clinical Modification* (ICD-9-CM) discharge diagnosis code of 779.5 (drug withdrawal syndrome in a newborn) or 760.72 (narcotics affecting fetus or newborn via placenta or breast milk) were considered to have possible NAS. NICU staff provided the investigation team with a list of infants admitted to the NICU for NAS treatment, based on documentation in NICU admission logs. Additionally, inpatient pharmacy dispensing data were used to identify infants treated with morphine, methadone, or clonidine during the 2-year period.

Infant and maternal medical records were abstracted. Infants meeting all three of the following criteria were classified as having confirmed NAS (hereafter referred to as NAS): 1) presence of a constellation of clinical signs consistent with NAS (defined as a documented NAS score >8 [on a scale of 0–37]) (3), not explained by another etiology; 2) documented history of maternal use during pregnancy of prescription or illicit drugs associated with NAS (1) or laboratory confirmation of recent maternal drug use or fetal exposure to such drugs; and 3) a severity of illness that resulted in a prolonged (>2 days) neonatal hospitalization. Descriptive statistics for infants with NAS were calculated by comparing infant data abstracted from medical records with data obtained from the linked administrative data on all infants (excluding medical record numbers of infants with NAS) born at the participating hospitals during the 2-year period. Z-tests were used to compare population proportions, and t-tests were used to compare means.

The linked administrative data identified 179 infants with ICD-9-CM codes 779.5 or 760.72. An additional 234 unique infants were identified from the NICU and pharmacy data, for a total of 413 infants with possible NAS whose medical records were reviewed, along with their mother’s medical record (when available). Of the 413 infants, 242 infants were classified as having NAS. There were 22,285 infants without NAS identified in the linked administrative data.

The mean age of mothers of infants with NAS was slightly younger, at 27.4 years, compared with 28.2 years for mothers of infants without NAS (p=0.01) (Table 1). Most of the infants with NAS (82.6%) were non-Hispanic white, compared with 56.7% of infants without NAS (p<0.01). There was a significantly higher percentage of low birth weight (<2500 grams; 19.4% versus 8.0%) and preterm (<37 weeks gestation) delivery (18.2% versus 12.3%) among infants with NAS compared with infants without NAS. Almost all infants with NAS (97.1%) were admitted to the NICU, compared with 6.2% of infants without NAS. None of the infants with NAS died during their birth hospitalization.

The mean of the first documented NAS score >8 was 11.5 (Table 2). Urine toxicology screens were the most common type of screen performed on infants with NAS, with 86.4% of infants with NAS screened for substance exposure. Pharmacologic therapy to control signs of NAS was used in 89.7% of infants, with morphine being the most commonly selected treatment (used in 87.6% of cases), followed by phenobarbital (used in 36.8% of cases). The mean NICU length of stay for infants with NAS was 26.1 days, and their mean age at discharge was 27.4 days. At discharge, most infants with NAS were receiving formula only (94.6%), approximately 4% were receiving both breast milk and formula, and none were documented as being exclusively breastfed.

There was documentation in the medical records of opioid use during pregnancy for nearly all (99.6%) mothers of infants with NAS. Approximately 82% of mothers were reported as using one or more opioid such as oxycodone, morphine, hydrocodone, hydromorphone, tramadol, or meperidine; 59.9% as using methadone; and 3.7% as using buprenorphine. Less than 1% of mothers were reported to have used heroin during pregnancy. Benzodiazepines were the second most commonly reported substances used (40.5%), followed by tobacco (39.7%), marijuana (24.4%), and cocaine (14.1%). Reasons reported for opioid use included illicit (i.e., nonmedical) (55.0%), drug abuse treatment (41.3%), and chronic pain treatment (21.5%). The reason for opioid use during pregnancy was unknown for 10.3% of NAS mothers. Urine toxicology screens were performed on 86.8% of the mothers of infants with NAS; of these, 90.5% had positive urine screen results. Lastly, 10.3% of mothers had documentation in the medical records that they had received or were referred for drug addiction rehabilitation or counseling during the infant’s birth hospitalization.

## Discussion

Infants with NAS have prolonged hospital stays, they experience serious medical complications, and their treatment is very costly (2,4). Overall, 242 infants with NAS were identified in this investigation of three Florida hospitals over a 2-year period. The majority of these infants were admitted to the NICU, where the mean length of stay was 26.1 days. Nearly all infants with NAS were exposed to opioids in utero (99.6%), highlighting the issue of opioid use in women of childbearing age (5). Additionally, it has been reported that women face many barriers in accessing any type of substance abuse treatment (6), which might also be reflected in the finding that only 10.3% of mothers of infants with NAS received or were referred for drug addiction rehabilitation or counseling during their infant’s birth hospitalization, despite a high percentage of mothers with positive urine toxicology screen results. Medication assisted treatment (MAT) is recommended as the standard of care for pregnant women with opioid addiction*; comprehensive MAT coupled with prenatal care can reduce complications associated with untreated opioid use disorder (1,7). None of the infants with NAS were documented to be exclusively breastfed at discharge, and only 3.7% of these infants were receiving any breast milk. There is some evidence that breastfeeding or the feeding of human milk might result in decreased intensity and severity of NAS (8–10), and current recommendations are that when possible, and not otherwise contraindicated, mothers in supervised drug treatment programs be encouraged to breastfeed (1).

The findings in this report are subject to at least five limitations. First, this investigation was conducted in three Florida hospitals and might not be representative of the state overall or other hospitals in Florida. Second, during the 2-year period, NAS scoring tools were not routinely included in electronic medical records at the participating hospitals; therefore, some infants with a NAS score >8 might have been missed if not documented somewhere in the medical record. Third, the hospitals were only able to provide pharmacy data based on the medication dispense date, not infant date of birth. Infants born near the end of 2011, but not dispensed pharmacologic treatment for NAS until 2012 might not have been included. Fourth, because NAS can be associated with a wide variety of pharmaceuticals and other substances and there was no “probable” case definition, the number of infants with NAS might be an underestimate of cases in the participating hospitals. Finally, only the feeding method on the day of discharge was collected; feeding methods during the infant’s birth hospitalization and reasons for not breastfeeding at discharge are unknown.

What is already known on this topic?

Infants with neonatal abstinence syndrome (NAS) have prolonged hospital stays, experience serious medical complications, and are very costly to treat.

What is added by this report?

During a 2-year period (2010–2011), a total of 242 confirmed NAS cases were identified in three Florida hospitals. Nearly all infants with NAS (99.6%) were exposed to opioids during pregnancy and experienced serious medical complications, with 97.1% being admitted to an intensive care unit, where the mean length of stay was 26.1 days.

What are the implications for public health practice?

Interventions are needed to 1) increase the number and use of community resources available to drug-abusing and drug- dependent women of reproductive age, 2) improve drug addiction counseling and rehabilitation referral and documentation policies, and 3) link women to these resources before or earlier in pregnancy. Encouraging breastfeeding of infants with NAS, when mothers are in supervised drug treatment programs and when not otherwise contraindicated, might also be considered.

Other analyses from this investigation will evaluate the use of Florida’s linked administrative data for NAS surveillance. The findings of this report enhance current clinical and public health knowledge on infants with NAS. When not otherwise contraindicated, encouraging mothers in supervised drug treatment programs to breastfeed, increasing the number and use of community resources available to women of reproductive age for substance abuse treatment and smoking cessation, improving drug addiction counseling and rehabilitation referral and documentation policies, and linking women to these resources before or earlier in pregnancy are all options that should be considered when addressing NAS prevention and management measures.

## Figures and Tables

**TABLE 1:** Selected characteristics of infants with confirmed neonatal abstinence syndrome (NAS) compared with infants without NAS — selected hospitals in Florida, 2010–2011

Characteristic	Confirmed NAS* (N = 242)		Non-NAS† (N = 22,285)		p-value§
	
	No.	(%)	No.	(%)	
**Mother’s age (yrs), mean ±SD**	**27.4 ±4.9**		**28.2 ±6.1**		**0.01**
**Sex**					
Male	136	(56.2)	11,466	(51.5)	0.14
Female	106	(43.8)	10,819	(48.6)	0.14
**Race/Ethnicity**					
White, non-Hispanic	200	(82.6)	12,645	(56.7)	<0.01
Black, non-Hispanic	3	(1.2)	3,851	(17.3)	<0.01
Hispanic	18	(7.4)	4,031	(18.1)	<0.01
Other	13	(5.4)	1,729	(7.8)	0.10
Unknown/Missing	8	(3.3)	29	(0.1)	0.01
**Birth weight**					
<2500 grams (low)	47	(19.4)	1,785	(8.0)	<0.01
≥2500 grams (normal)	195	(80.6)	20,500	(92.0)	<0.01
**Gestational age**					
<37 weeks (preterm)	44	(18.2)	2,730	(12.3)	0.02
≥37 weeks (term)	198	(81.8)	19,551	(87.8)	0.02
**5-minute APGAR score, mean ±SD**	**8.8 ±0.6**		**8.9 ±0.6**		**0.06**
**NICU admission**					
Yes	235	(97.1)	1,386	(6.2)	<0.01
No	7	(2.9)	20,899	(93.8)	<0.01
**Infant death**					
Yes	0	(0.0)	103	(0.5)	<0.01
No	242	(100.0)	22,182	(99.5)	<0.01

**Abbreviations:** NICU = neonatal intensive care unit; SD = standard deviation.

* Case definition of confirmed NAS, based on hospital medical record abstraction; all three of the following conditions must be met: 1) presence of a constellation of clinical signs consistent with NAS, not explained by another etiology; 2) documented history of maternal use of prescription or illicit drugs normally associated with NAS during pregnancy and/or laboratory confirmation of recent maternal drug use or fetal exposure to such drugs; and 3) a level of severity of signs that result in a neonatal hospitalization beyond the first few days of life (defined as a hospital stay >2 days).

† Data on the infants without NAS were obtained from Florida’s linked administrative data and includes all births at the selected hospitals during 2010–2011, excluding infants with confirmed NAS.

§ Z-tests were used to compare population proportions. T-tests were used to compare means.

**TABLE 2:** Selected characteristics of infants with confirmed neonatal abstinence syndrome (NAS) and their mothers — selected hospitals in Florida, 2010–2011

Infant characteristics	Confirmed NAS* (N = 242)	

	No.	(%)
**First documented NAS score >8, mean ±SD** †	**11.5 ±2.6**	
**Toxicology screens performed** §		
Urine	209	(86.4)
Meconium	74	(30.6)
Umbilical cord tissue	63	(26.0)
**Pharmacologic therapy used for NAS** §		
Any type of pharmacologic therapy	217	(89.7)
Morphine sulfate	212	(87.6)
Phenobarbital	89	(36.8)
Clonidine	9	(3.7)
Methadone	3	(1.2)
Midazolam	2	(0.8)
Fentanyl	2	(0.8)
Chloral hydrate	1	(0.4)
**NICU length of stay (days), mean ±SD**	**26.1 ±15.3**	
**Age at discharge (days), mean ±SD**	**27.4 ±15.6**	
**Feeding methods on day of discharge**		
Breastfeeding only	0	(0.0)
Formula only	229	(94.6)
Mixed breastfeeding and formula	9	(3.7)
Other/Unknown	4	(1.7)

Maternal characteristics	Confirmed NAS* (N = 242)	

	No.	(%)
**Substances used during pregnancy** §		
Opioids	241	(99.6)
Other opioids¶	198	(81.8)
Methadone	145	(59.9)
Buprenorphine	9	(3.7)
Heroin	2	(0.8)
Benzodiazepines	98	(40.5)
Tobacco	96	(39.7)
Marijuana/Hashish	59	(24.4)
Cocaine	34	(14.1)
Antidepressants	17	(7.0)
Other	16	(6.6)
Barbiturates	12	(5.0)
Methamphetamine	8	(3.3)
Other amphetamines/CNS stimulants	8	(3.3)
Alcohol	5	(2.1)
Other sedative-hypnotics	2	(0.8)
**Reasons for opioid use** §		
Illicit	133	(55.0)
Drug abuse treatment	100	(41.3)
Chronic pain	52	(21.5)
Unknown	25	(10.3)
**Urine toxicology screen performed**		
Yes	210	(86.8)
No/Unknown	32	(13.2)
**Positive urine toxicology screen**		
Yes	190	(90.5)
No/Unknown	20	(9.5)
**Services received during birth hospitalization** §		
Referral for drug addiction rehabilitation	15	(6.2)
Drug addiction counseling/Counseling on substance use and abuse	10	(4.1)

**Abbreviations:** NICU = neonatal intensive care unit; SD = standard deviation; CNS = central nervous system.

* Case definition of confirmed NAS, based on hospital medical record abstraction; all three of the following conditions must be met: 1) presence of a constellation of clinical signs consistent with NAS, not explained by another etiology; 2) documented history of maternal use of prescription or illicit drugs normally associated with NAS during pregnancy and/or laboratory confirmation of recent maternal drug use or fetal exposure to such drugs; and 3) a level of severity of signs that result in a neonatal hospitalization beyond the first few days of life (defined as a hospital stay >2 days).

† Scores can range from 0 to 37; scores >8 are typically considered indicative of NAS.

§ More than one response possible; therefore, percentages might not sum to 100%.

¶ Including oxycodone, morphine, hydrocodone, hydromorphone, tramadol, and meperidine.

## Footnotes

* Additional information on substance use disorder during pregnancy is available at http://www.samhsa.gov.
